# Dual-stream cross-modal fusion alignment network for survival analysis

**DOI:** 10.1093/bib/bbaf103

**Published:** 2025-03-21

**Authors:** Jinmiao Song, Yatong Hao, Shuang Zhao, Peng Zhang, Qilin Feng, Qiguo Dai, Xiaodong Duan

**Affiliations:** School of Software, Xinjiang University, Urumqi 830046, China; School of Computer Science and Engineering, Dalian Minzu University, Dalian 116650, China; State Ethnic Affairs Commission Key Laboratory of Big Data Applied Technology, Dalian Minzu University, Dalian 116650, China; Dalian Key Laboratory of Digital Technology for Minzu Culture, Dalian Minzu University, Dalian 116650, China; School of Computer Science and Engineering, Dalian Minzu University, Dalian 116650, China; State Ethnic Affairs Commission Key Laboratory of Big Data Applied Technology, Dalian Minzu University, Dalian 116650, China; Dalian Key Laboratory of Digital Technology for Minzu Culture, Dalian Minzu University, Dalian 116650, China; School of Software, Xinjiang University, Urumqi 830046, China; School of Software, Xinjiang University, Urumqi 830046, China; School of Computer Science and Engineering, Dalian Minzu University, Dalian 116650, China; State Ethnic Affairs Commission Key Laboratory of Big Data Applied Technology, Dalian Minzu University, Dalian 116650, China; Dalian Key Laboratory of Digital Technology for Minzu Culture, Dalian Minzu University, Dalian 116650, China; School of Computer Science and Engineering, Dalian Minzu University, Dalian 116650, China; State Ethnic Affairs Commission Key Laboratory of Big Data Applied Technology, Dalian Minzu University, Dalian 116650, China; Dalian Key Laboratory of Digital Technology for Minzu Culture, Dalian Minzu University, Dalian 116650, China

**Keywords:** whole slide image, cancer diagnosis, multimodal learning, survival prediction

## Abstract

Survival prediction serves as a pivotal component in precision oncology, enabling the optimization of treatment strategies through mortality risk assessment. While the integration of histopathological images and genomic profiles offers enhanced potential for patient stratification, existing methodologies are constrained by two fundamental limitations: (i) insufficient attention to fine-grained local features in favor of global representations, and (ii) suboptimal cross-modal fusion strategies that either neglect intrinsic correlations or discard modality-specific information. To address these challenges, we propose DSCASurv, a novel cross-modal fusion alignment framework designed to explore and integrate intrinsic correlations across multimodal data, thereby improving the accuracy of survival prediction. Specifically, DSCASurv leverages the local feature extraction capabilities of convolutional layers and the long-range dependency modeling of scanning state space models to extract intra-modal representations, while generating cross-modal representations through dual parallel mixer architectures. A cross-modal attention module functions as a bridge for inter-modal information exchange and complementary information transfer. The framework ultimately integrates all intra-modal representations to generate survival predictions by enhancing and recalibrating complementary information. Extensive experiments on five benchmark cancer datasets demonstrate the superior performance of our approach compared to existing methods.

## Introduction

Survival prediction is crucial in cancer treatment. By analyzing patients’ genetic, transcriptomic, or other types of data, statistical models or deep learning models are utilized to predict disease progression and survival probabilities [1]. Traditional survival analysis relies on clinical indicators and follow-up reports, but advancements in deep learning technology have made medical image analysis more efficient. Pathological images, especially whole slide imaging (WSI) at gigapixel resolution, showcase the tumor microenvironment, capturing the diversity of cancer cells and immune interactions, which has significant potential to enhance the accuracy of cancer survival predictions [2, 3]. However, unlike clearly labeled natural images, WSI presents significant heterogeneity as a large and complex type of visual data, and survival predictions often depend on delicate visual features and interactions among different regions [4].

Due to the enormous gigapixel resolution of WSI, researchers have introduced multiple instance learning (MIL) methods to achieve effective representation learning for WSIs. For instance, Jiang et al. [5]proposed a deep learning-based risk scoring method that directly extracts risk scores for colorectal cancer prognosis from WSIs. Zhu et al. [6]developed a framework called WSISA, which employs an adaptive sampling strategy to extract a large number of candidate image patches from WSIs and clusters them for patient-level predictions in small sample datasets. Although these methods utilize adaptive sampling strategies that help cover a broader range of image areas, they may not be sufficiently precise in handling local microfeatures, which can subsequently affect prediction accuracy. This is particularly critical in tumor tissues, where local structural differences often have a significant impact on survival outcomes. In contrast, Xu et al. [7] proposed a survival time prediction method based on graph convolutional networks (GCNs). This approach extracts tumor patches from WSIs, screens them using a pre-trained tumor classifier, and aggregates information through GCNs. However, despite combining risk prediction and time prediction strategies to enhance accuracy, this method still exhibits shortcomings in modeling and utilizing local features. Specifically, when processing high-resolution WSIs, it primarily relies on global features aggregation, which may overlook the subtle interactions between genotype and phenotype in the tumor microenvironment, thereby limiting its precision in survival predictions. Thus, while these methods have made significant advancements in various aspects, they still demonstrate certain limitations in capturing and utilizing local features, indicating the necessity to develop a novel model that better balances the use of long-range information and local features.

Although traditional survival analysis models focus on a single data source, integrating genomic information into the analysis of pathological images can provide additional supervision or enriched multimodal features, thereby enhancing the prediction of patient survival outcomes. Currently, multimodal learning methods based on genomic features and pathological images can be broadly categorized into two approaches: the first approach directly integrates all features learned from multimodal data for survival prediction; the second approach uses genomic features to guide the integration of pathological image features, allowing the model to focus on parts of the pathological images relevant to gene expression. For instance, Sun et al. [8] combined genomic data with pathological image features through a multiple kernel learning (MKL) approach to construct the GPMKL model, achieving effective integration and application of multimodal data in breast cancer survival prediction. Additionally, MCAT [9] proposed a Transformer-based multimodal co-attention model that combines gigapixel-level WSIs with genomic features and employs the attention mechanism of Transformers for feature aggregation, facilitating interactions between modalities. However, these methods still have limitations. The former approach, when integrating pathological images and genomic data, only performs simple fusion without thoroughly exploring the potential associations and interactions between modalities, leading to insufficient cross-modal information integration. While the latter, MCAT emphasizes image regions related to gene expression, it may overlook pathological information that, not directly related to gene expression, it still holds significant diagnostic value, thus failing to fully leverage all information within the pathological images. Furthermore, these methods primarily focus on intra-modal alignment, neglecting inter-modal alignment, which may result in the loss of important information during the multimodal data fusion process, subsequently affecting the overall performance of the model. Hence, future research should place greater emphasis on how to effectively achieve alignment and fusion between modalities to maximize the complementary information of multimodal data, thereby improving the accuracy and reliability of survival analysis.

Based on these observations, we propose a novel cross-modal fusion alignment framework (DSCASurv) to explore the intrinsic cross-modal correlations and convey complementary information. Specifically, we construct two parallel mixer structures that combine the local feature extraction capability of convolutional layers with the long-range modeling ability of SSM, allowing us to extract intra-modal representations from single modalities and generate cross-modal representations from inter-modal information. To investigate the potential correlations between modalities, we utilize a cross-modal attention module as an information bridge between different modalities, facilitating cross-modal interactions and the transfer of complementary information. This approach enhances and recalibrates the complementary information by leveraging both cross-modal and intra-modal representations. Finally, we integrate all intra-modal representations to produce the final survival prediction. Extensive experiments conducted on five public The Cancer Genome Atlas (TCGA) datasets validate the effectiveness of the proposed model. Results demonstrate superior performance compared to state-of-the-art methods.

## Related works

### Survival prediction from single modality

Survival risk prediction plays a crucial role in assessing cancer progression. Radiology, as a traditional unimodal survival analysis method [10, 11], primarily extracts quantitative features from imaging data using radiological techniques. Compared to radiology, pathology has demonstrated greater potential in survival prediction. Liu et al. [12] developed the Advmil model based on an adversarial MIL framework, which enhances the identification of key regions in pathological images. With the development of high-throughput sequencing technologies, genomic data have become increasingly important. Mosquera Orgueira et al. [13] explored a novel survival model based on machine learning using transcriptomic, demonstrating that gene expression profiling significantly improves the accuracy. However, relying solely on single-modality data (radiological data, pathological data, genomic data) is often unable to capture all the characteristics of complex diseases. To overcome this limitation, recent studies have increasingly shifted toward multimodal survival prediction [14, 15].

### Survival prediction from multiple modalities

Different modalities(such as genomics, pathology, and radiology data) each offer unique advantages, and by integrating these data,thereby optimizing diagnosis and treatment plans [16]. In the research process of multimodal survival prediction, the initial work mainly focused on simple splicing or weighted summation of features from different modalities [17]. But this approach overlooks the potential interactive information among modalities. As research in multimodal survival prediction advanced, increasingly complex fusion methods were developed to optimize the integration of information between different modalities and improve prediction precision. The TTMFN model proposed by Ge et al. [18] adopts a dual-stream Transformer architecture, this model not only effectively captures intra-modality relationships but also explores complementarities between modalities. Additionally, Vollmer et al. [19] integrated pathology data, genomic data, and clinical information, this multimodal fusion approach overcome the limitations of single data types, and thus enhances the overall performance of predictive models. Guo et al.[20] proposed a graph-based fusion method that integrates imaging, genetic, and clinical data for the diagnosis of degenerative diseases through an imaging-genetic fusion module and a multi-graph fusion module. However, these methods may lead to loss or redundancy of modality-specific information during cross-modal interaction [21, 22]. Therefore, our model employs a cross-modality fusion alignment framework, which not only preserves modality-specific information but also effectively integrates multimodal data, making a significant contribution to the advancement of multimodal survival prediction methods.

### Mamba structure

In recent years, the state space models (SSMs) have demonstrated significant advantages in handling dynamic systems and modeling long-range dependencies, and have been widely used in medical image analysis in particular [23]. Gu et al. [24] introduced a method called Mamba for linear time series modeling. This approach leverages a selective state space to effectively capture long-range dependencies in sequential data. Subsequently, the MedMamba model proposed by Yue et al. [25] significantly improves the accuracy of medical image classification through a structured multimodal data processing mechanism. Similarly, the SurvMamba model introduced by Chen et al. [2] effectively enhances the performance of survival prediction models through a multilevel, multimodal interaction mechanism. Overall, the application of Mamba models in medical imaging analysis showcases the tremendous potential of SSMs. Thus, we constructed two parallel mixer structures that combine the local feature extraction capabilities of convolutional layers with the long-range modeling capabilities of SSMs, extracting intra-modality representations within a single modality and generating cross-modality representations from cross-modality information.

## Methodology

**Figure 1:**
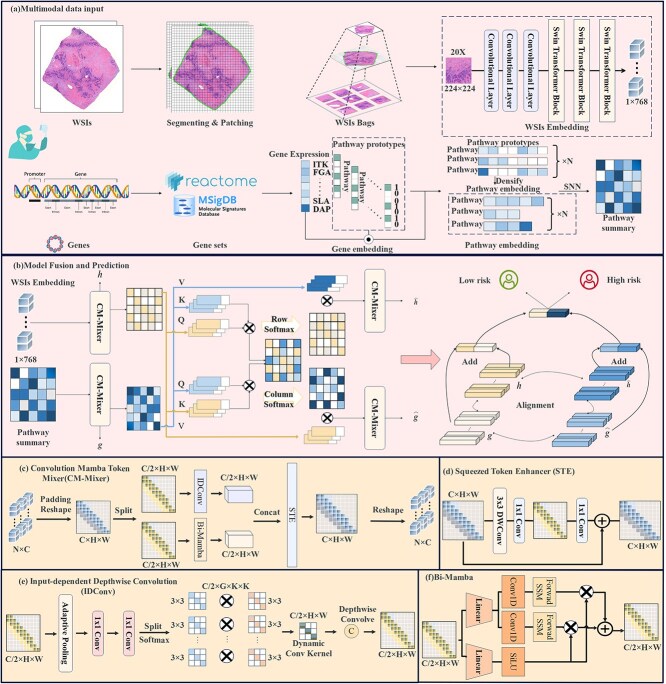
Framework diagram of the DSCASurv model.(a) The multimodal feature input module extracts features from gene and pathological data.(b) The fusion and prediction model of the model outputs high-risk and low-risk values.(c) We propose the CM-Mixer, which aggregates sparse global information and local details in a context-sensitive manner, generating strong inductive biases that enhance the model’s generalization capability.(d), (e), (f) Key components of the CM-Mixer.

In this section, we first describe the components of an SSM, and then outline our proposed DSCASurv and its core components, as shown in Fig. 1.

### Preliminaries

Recent models based on SSMs, such as structured state space sequence models (S4) and Mamba, rely on a classical continuous system that maps a 1D input function or sequence, denoted as $ x(t) \in \mathbb{R} $, through intermediate latent states $ h(t) \in \mathbb{R}^{N} $, to an output $ y(t) \in \mathbb{R} $. This process can be expressed as a linear ordinary differential equation as


(1) \begin{align*} & h^{\prime}(t) = Dh(t) + Ex(t) \end{align*}

(2) \begin{align*} & y(t) = Fh(t)\qquad\quad \end{align*}

where $ D \in \mathbb{R}^{N \times N} $ represents the state matrix, and $ E \in \mathbb{R}^{N \times 1} $ and $ F \in \mathbb{R}^{N \times 1} $ represent the projection parameters.

The S4 model and Mamba discretize this continuous system to adapt it to deep learning applications. Specifically, they introduce a timescale parameter $ \Delta $, which is used to transform $ D $ and $ E $ into discrete parameters $ \bar{D} $ and $ \bar{E} $ by applying a fixed discretization rule. Typically, the zero-order hold [26] is employed as the discretization method, which can be defined as


(3) \begin{align*} & \bar{D} = exp(\Delta D) \quad\qquad\end{align*}

(4) \begin{align*} & \qquad\qquad\ \ \ \ \bar{E} = (\Delta D)^{-1} (exp(\Delta D) - I) \cdot \Delta E \end{align*}

Once the system is discretized with a step size $ \Delta $, Equations 1 and 2 can be rewritten as


(5) \begin{align*} & h^{\prime}(t) = \bar{D}h(t) + \bar{E}x(t) \end{align*}

(6) \begin{align*} & y(t) = Fh(t)\qquad\quad\ \end{align*}

Finally, the SSM model utilizes a global convolution to compute the output as shown:


(7) \begin{align*} &\qquad \bar{K} = (F \bar{E}, F \bar{D} \bar{E},..., F \bar{D}^{M-1} \bar{E}) \end{align*}

(8) \begin{align*} & y = x * \bar{K}\qquad\qquad\quad \end{align*}

### Problem formulation

Let $ Z = \{ Z_{1}, Z_{2}, \cdots , Z_{X} \} $ represent the clinical data of $ X $ patients. Each patient’s data can be represented by a quadruple $Z_{i} = (P_{i}, G_{i}, s_{i}, t_{i})$, where $ P_{i}\in \mathbb{R}^{N\times d} $ is the set of whole slide images, $ G_{i}\in \mathbb{R}^{K\times d} $ is the set of genomic profiles, $N$ is the number of patches, $K$ is the number of biological pathways, the dimension $d$ represents the matching dimension between the genomic and pathological features, and $ s_{i} \in \{0, 1\} $ represents the correct censoring status, with $ s_{i} = 0 $ indicating the observed death of the patient, and $ s_{i} = 1 $ corresponding to the patient’s last known follow-up. The $ t_{i} \in{\mathbb{R}}^{+} $ is the event time. If $ s_{i} = 0 $, $ t_{i} $ corresponds to the time between diagnosis and the observed death of the patient; if $ s_{i} = 1 $, $ t_{i} $ corresponds to the time of the last follow-up. Our goal is to predict patient survival outcomes by estimating the hazard function $ f_{\text{hazard}}(t) $, which represents the probability of death at time point $ t_{i} $.

In survival analysis, $T$ represents the random variable for total survival time. Our objective is to create a survival prediction model $H$ that integrates the multimodal data, $P$ and $G$, to estimate the hazard function $f_{\text{hazard}}(T = t)$, as expressed as


(9) \begin{align*} & f_{\text{hazard}}(T = t) = \lim_{\partial t \to 0} \frac{P(t \leq T \leq t + \partial t | T \geq t)}{\partial t}\end{align*}

The hazard function gives the instantaneous rate at which the event of interest (e.g. death) occurs at time $t$. In practice, we estimate the probability that a patient survives past a set of discrete time points rather than estimating the exact survival time. The survival function can be derived from the cumulative hazard function as follows:


(10) \begin{align*} & f_{{surv}}(T \leq t, Z) = \prod_{u=1}^{t} \left(1 - f_{\text{hazard}}^{i}(T = u)\right)\end{align*}

### CM-Mixer

To efficiently leverage both global and local information, this introduce a lightweight token mixer, the Convolutional Mamba Token Mixer (CM-Mixer). This module incorporates inductive biases, thereby enhancing the model’s generalization capability without compromising its dependency on input features. Figure 1c illustrates the overall architecture of CM-Mixer. Specifically, for an input feature map $F \in \mathbb{R}^{C \times H \times W}$, we first divide it along the channel dimension into two sub-feature maps $\lbrace F1, F2 \rbrace \in \mathbb{R}^{C/2 \times H \times W}$. The sub-features $F_{1}$ and $F_{2}$ are subsequently passed through Bi-Mamba [27] and a dynamic depthwise convolution, IDConv [28], producing new feature maps $\lbrace F^{\prime}1, F^{\prime}2 \rbrace \in \mathbb{R}^{C/2 \times H \times W}$. Then these are concatenated along the channel dimension, resulting in the output feature map $F^{\prime} \in \mathbb{R}^{C \times H \times W}$. Finally, a Shrinking Token Enhancer (STE) is applied to achieve efficient local token aggregation. The CM-Mixer can be represented by the following steps:


(11) \begin{align*} & \begin{array}{l} \lbrace{F}_{1}, {F}_{2} \rbrace= \operatorname{Split}({F}) \\{F}^{\prime} = \operatorname{Concat}(\operatorname{Bi-Mamba}({F}_{1}), \operatorname{IDConv}({F}_{2})) \\ Y = \operatorname{STE}(F^{\prime}) \end{array}\end{align*}

As shown, the dynamic feature aggregation weights generated by Bi-Mamba and IDConv encapsulate both global and local details, allowing the model to perform powerful representation learning.

**1) Bi-Mamba:** The Bi-Mamba architecture, as depicted in Fig. 1f, begins by linearly projecting the input token sequence $T_{l-1}: (B, M, D)$ into two feature representations, $\mathbf{x}$ and $\mathbf{z}$. For each direction $o \in \{\text{forward}, \text{backward}\}$, the algorithm applies a 1D convolution followed by a SILU activation to $\mathbf{x}$, producing $\mathbf{x}^{\prime }_{o}$. Subsequent linear projections generate $B_{o}$, $C_{o}$, and $\Delta _{o}$, which are utilized to transform the parameters $A_{o}$ and $B_{o}$ into $\overline{A_{o}}$ and $\overline{B_{o}}$. The SSM is then employed to compute the directional outputs $Y_{o}$. These outputs are gated using $\mathbf{z}$, and the resulting $Y_{\text{forward}}$ and $Y_{\text{backward}}$ are combined, linearly transformed, and integrated to produce the final output $T_{l}$. This approach effectively captures bidirectional dependencies while preserving critical information through efficient processing.

**2) Input-dependent depthwise convolution:** To induce local feature aggregation in an input-adaptive manner, we introduce IDConv as a key component of CM-Mixer, depicted in Fig. 1e. Given an input feature map $F \in \mathbb{R}^{C \times H \times W}$, adaptive average pooling is applied to compress the spatial dimensions to $M^{2}$. This compressed map is then passed through two sequential $1 \times 1$ convolutions, generating an attention map ${G}^{\prime} \in \mathbb{R}^{(A \times C) \times M^{2}}$, where $A$ is the number of attention groups. The map ${G}^{\prime}$ is reshaped into $\mathbb{R}^{(A \times C) \times M^{2}}$, and a softmax function is applied along the $A$ dimension to produce attention weights $G \in \mathbb{R}^{(A \times C) \times M^{2}}$. The attention weights $G$ are then element-wise multiplied by a set of learnable parameters $Q \in \mathbb{R}^{(A \times C) \times M^{2}}$, and the result is summed over $A$ to obtain the input-dependent convolutional kernel $W \in \mathbb{R}^{C \times M^{2}}$ as follows:


(12) \begin{align*} & \begin{array}{l} G^{\prime} = \operatorname{Conv}_{1 \times 1}^{\frac{C}{r} \rightarrow (A \times C)}(\operatorname{Conv}_{1 \times 1}^{C \rightarrow \frac{C}{r}}(\operatorname{AdaptivePool}(F))) \\ G = \operatorname{Softmax}(\operatorname{Reshape}(G^{\prime})) \\ W = \sum_{j=0}^{A} Q_{j} G_{j} \end{array}\end{align*}

**3) Squeezed Token Enhancer (STE):** To minimize computational complexity while preserving performance, we employ a lightweight STE, shown in Fig. 1d. The STE consists of a $3 \times 3$ depthwise convolution to capture local relationships, followed by a $1 \times 1$ convolution for channel expansion and compression, reducing computation costs. A residual connection is also used to maintain representational integrity. The STE operation is expressed as


(13) \begin{align*} & \operatorname{STE}(F^{\prime}) = \operatorname{Conv}_{1 \times 1}^{\frac{C}{r} \rightarrow C}(\operatorname{Conv}_{1 \times 1}^{C \rightarrow \frac{C}{r}}(\operatorname{DWConv}_{3 \times 3}(F^{\prime}))) + F^{\prime}\end{align*}

### Cross-modal attention module

In this section, we introduce two co-attention mechanisms: genomic-guided co-attention (GCA) and pathology-guided co-attention (PCA). These mechanisms enable us to explore potential cross-modal relationships and facilitate the transfer of complementary information between multimodal data.

**1) Pathology-guided co-attention:** The PCA leverages pathology information to guide the aggregation of genomic features, transforming them into a set of pathology-guided visual cluster representations. Specifically, we define three linear projections of the tokens using learnable matrices: $ W_{Q}^{p} \in \mathbb{R}^{d \times d_{q}}, W_{K}^{g} \in \mathbb{R}^{d \times d_{k}}, \text{and} W_{V}^{g} \in \mathbb{R}^{d \times d_{v}} $ to extract the $Q_{p}$, $K_{g}$, and $V_{g}$. The attention mechanism is then given by


(14) \begin{align*} & CoAttn_{PCA} = \operatorname{softmax}\left( \frac{Q_{p} \times K_{g}^{T}}{\sqrt{d}} \right) V_{g}\end{align*}

**2) Genomic-guided co-attention:** The GCA utilizes genomic information to guide the aggregation of pathology features, transforming them into a set of genomic-guided visual cluster representations. We define three linear projections of the tokens using learnable matrices: $ W_{Q}^{g} \in \mathbb{R}^{d \times d_{q}}, W_{K}^{p} \in \mathbb{R}^{d \times d_{k}}, \text{and} W_{V}^{p} \in \mathbb{R}^{d \times d_{v}} $ to extract the $Q_{g}$, $K_{p}$, and $V_{p}$. The attention mechanism is given by


(15) \begin{align*} & CoAttn_{GCA} = \operatorname{softmax}\left( \frac{Q_{g} \times K_{p}^{T}}{\sqrt{d}} \right) V_{p}\end{align*}

### Feature alignment and fusion

Cross-modal representations can provide complementary information that may not be visible in a single modality. Therefore, we leverage both cross-modal and intra-modal representations to enhance and recalibrate complementary information representations. First, cross-modal and intra-modal features are globally averaged pooling, and then all feature representations are integrated to produce the final survival prediction. Feature fusion and survival prediction can be formulated as


(16) \begin{align*} & T_{1}, \cdots, T_{t} = \text{sigmoid}\left( \text{MLP} \left( h + \hat{h} + g + \hat{g} \right) \right)\end{align*}

In practice, professional pathologists and biologists can estimate partial gene expression from pathological images or imagine possible pathological phenotypes from genomic maps. Our mixer is designed to obtain cross-modal public information. In order to ensure the quality of public information, we must impose alignment constraints. This paper uses the $L_{1}$ norm to measure the distance between cross-modal representation and intra-modal representation.


(17) \begin{align*} & \mathcal{L}_{\text{sim}} = \frac{1}{d} \left( \|h - \hat{h}\| + \|g - \hat{g}\| + \|h - g\| + \|\hat{h} - \hat{g}\| \right)\end{align*}

We use negative log likelihood [29] survival loss as the loss function for the survival prediction part. Unifying these two losses, we can get the total loss function of DSCASurv framework:


(18) \begin{align*} & \mathcal{L}_{ {total}} =\mathcal{L}_{ {sur }}+\alpha \mathcal{L}_{ {sim }}\end{align*}

where $\alpha $ is a positive hyperparameter used to moderate the contribution to the alignment loss function.

## Experimental

In this section, we provide an in-depth description of the adopted dataset and its characteristics, and clearly define the criteria for evaluating the performance of the model. Experimental results show that our proposed method not only outperforms existing techniques in terms of prediction accuracy, but also demonstrates significant advantages in terms of computational efficiency. In order to further verify the validity and reliability of the method in the field of survival analysis, we introduce advanced statistical methods such as Kaplan–Meier survival curve analysis and log-rank test.

### Datasets and implementation details

**Datasets.** Our study utilized five TCGA cancer cohorts: breast invasive carcinoma (BRCA, n=869), gastric adenocarcinoma (STAD, n=317), bladder urothelial carcinoma (BLCA, n=359), and head-neck squamous cell carcinoma (HNSC, n=392), colorectal adenocarcinoma (COADREAD, n=296). Table 1 summarizes key dataset characteristics, where “Time” denotes maximum follow-up months and “Censored” represents the proportion of censored patients in the dataset. Building upon Jaume et al.’s framework [15], we developed an optimized disease-specific survival prediction model incorporating two multimodal inputs: (i) histopathological analysis primarily utilizing WSI from initial diagnoses; (ii) genomic profiling through the Xena platform following Qu et al.’s methodology [30]. Pathway selection adhered to stringent criteria requiring ¿90% transcriptomic data coverage across entries from the Molecular Signatures Database [31] and Reactome [32], ensuring analytical reliability. A weighted sampling approach effectively addressed class imbalance during model training.

**Table 1:** Dataset statistics across different cancer types

**Cancer type**	**Patient**	**Censored**	**Time**
BLCA	359	0.678	165.6
BRCA	869	0.933	99.7
COADREAD	296	0.877	150.0
HNSC	392	0.702	213.9
STAD	317	0.737	118.0

**Evaluation metrics.** We employed a 5-fold cross-validation strategy to evaluate the performance across each dataset. In this process, the concordance index (C-index) [33] and its standard deviation (STD) were used as quantitative metrics. We also plotted Kaplan–Meier survival curves to visualize the differences in survival probabilities among patients across various risk groups. Additionally, a log-rank test for statistical significance, following the method of Darmawan et al. [34], was performed to determine whether the separation between these groups was statistically significant.

**Implementation.** During WSI preprocessing, the primary task was the precise segmentation of tissue regions from complex backgrounds. Non-overlapping 224$\times $224 pixel image patches were extracted at 20$\times $ magnification, serving as the foundation for subsequent analysis. Feature extraction was performed using the Swin Transformer (CTransPath) [35], a self-supervised contrastive learning model pre-trained on over 14 million pan-cancer histopathology slides, producing 768-dimensional feature embeddings. Genomic data were organized into biological pathway groups (curated from MSIgDB Hallmarks and Reactome), with each pathway encoded into 256-dimensional embeddings using SNN, forming genomic bags. The framework was implemented in Python using PyTorch, with all computations performed on an NVIDIA V100 GPU. For survival prediction, overall survival time was discretized into four intervals, which were further classified into eight risk strata based on censoring status. To enhance data diversity, 4096 image patches were randomly sampled from each WSI as training samples. Optimization was carried out using the Adam optimizer [36], with a learning rate of $5 \times 10^{-4}$. All models, including comparison methods, were trained for 30 epochs with a batch size of 32. Model performance was evaluated using 5-fold cross-validation, reporting the mean and standard deviation of the C-index on the validation set.

### Comparisons with state-of-the-art

To thoroughly validate the performance of our approach, we not only implemented the most advanced state-of-the-art survival prediction techniques but also conducted a systematic evaluation and comparison. Table 2 provides a detailed summary of the experimental results for all methods across five TCGA datasets.

**Table 2:** Performance comparison of different methods on five TCGA datasets

**Model**	**g**	**p**	**COADREAD**	**BRCA**	**BLCA**	**HNSC**	**STAD**	**Overall**
MLP	*		$0.640 \pm 0.103$ ^†^	$0.639 \pm 0.130$ ^†^	0.583 $\pm $ 0.064	0.515 $\pm $ 0.072	$0.631 \pm 0.054$ ^†^	0.602
SNN	*		$0.660 \pm 0.111$ ^†^	$0.621 \pm 0.134$ ^†^	0.606 $\pm $ 0.077	0.590 $\pm $ 0.048	$0.652 \pm 0.035$ ^†^	0.626
SNNTrans	*		$0.737 \pm 0.110$ ^†^	$0.637 \pm 0.074$ ^†^	0.566 $\pm $ 0.022	0.599 $\pm $ 0.063	0.576 $\pm $ 0.032	0.642
ABMIL		*	$0.673 \pm 0.176$ ^†^	$0.676 \pm 0.085$ ^†^	0.569 $\pm $ 0.060	0.547 $\pm $ 0.067	0.505 $\pm $ 0.069	0.594
TransMIL		*	$0.759 \pm 0.108$ ^†^	$0.699 \pm 0.077$ ^†^	$0.628 \pm 0.035$ ^†^	$0.636 \pm 0.071$ ^†^	$0.647 \pm 0.107$ ^†^	0.674
MCAT(Cat)	*	*	$0.716 \pm 0.140$ ^†^	$0.740 \pm 0.036$ ^†^	$0.635 \pm 0.065$ ^†^	0.602 $\pm $ 0.079	$0.619 \pm 0.067$ ^†^	0.662
MCAT(KP)	*	*	$0.752 \pm 0.140$ ^†^	$0.725 \pm 0.061$ ^†^	$0.626 \pm 0.074$ ^†^	0.595 $\pm $ 0.039	$0.635 \pm 0.063$ ^†^	0.667
Porpoise(Cat)	*	*	$0.727 \pm 0.084$ ^†^	$0.687 \pm 0.105$ ^†^	$0.645 \pm 0.076$ ^†^	$0.630 \pm 0.058$ ^†^	$0.680 \pm 0.054$ ^†^	0.674
Porpoise(KP)	*	*	$0.725 \pm 0.135$ ^†^	$0.677 \pm 0.106$ ^†^	$0.656 \pm 0.062$ ^†^	$0.617 \pm 0.053$ ^†^	$0.674 \pm 0.048$ ^†^	0.670
MOTCAT(KP)	*	*	$\underline{0.780 \pm 0.098}$ ^†^	$0.733 \pm 0.112$ ^†^	$0.649 \pm 0.047$ ^†^	$0.622 \pm 0.063$ ^†^	$0.634 \pm 0.065$ ^†^	0.684
MOTCAT(Cat)	*	*	$0.765 \pm 0.157$ ^†^	$0.742 \pm 0.099$ ^†^	$0.651 \pm 0.048$ ^†^	$0.639 \pm 0.045$ ^†^	$0.660 \pm 0.066$ ^†^	0.691
CMTA(KP)	*	*	$0.776 \pm 0.141$ ^†^	$0.743 \pm 0.029$ ^†^	$0.655 \pm 0.050$ ^†^	$0.608 \pm 0.058$ ^†^	$0.635 \pm 0.052$ ^†^	0.683
CMTA(Cat)	*	*	$0.780 \pm 0.105$ ^†^	$\underline{0.749 \pm 0.091}$ ^†^	$\underline{0.670 \pm 0.056}$ ^†^	$0.629 \pm 0.058$ ^†^	$0.660 \pm 0.077$ ^†^	0.698
SurvPath	*	*	$0.733 \pm 0.139$ ^†^	$0.747 \pm 0.093$ ^†^	$\boldsymbol{0.671 \pm 0.072}$ ^†^	$0.625 \pm 0.074$ ^†^	$0.640 \pm 0.028$ ^†^	0.683
PIBD	*	*	$0.768 \pm 0.124$ ^†^	$0.736 \pm 0.072$ ^†^	$0.667 \pm 0.061$ ^†^	$\underline{0.640 \pm 0.039}$ ^†^	$\underline{0.684 \pm 0.035}$ ^†^	0.699
Ours	*	*	$\boldsymbol{0.832 \pm 0.095}$ ^†^	$\boldsymbol{0.765 \pm 0.061}$ ^†^	$0.646 \pm 0.034$ ^†^	$\boldsymbol{0.666 \pm 0.071}$ ^†^	$\boldsymbol{0.698 \pm 0.093}$ ^†^	**0.721**

(“$\dagger $” denotes *P*-value<0.05). The column “p” specifies if pathological images were utilized, while “g” denotes the inclusion of genomic profiles. The optimal outcomes are highlighted in bold, and the second-best results are underlined

**Unimodal baselines**. In the single-modality analysis, we respectively compared three methods: MLP, SNN [37], and SNNTrans [37]. In the field of histopathology, we further compared several leading MIL methods, including ABMIL [38] and TransMIL[39]. Our model has consistently performed outstandingly on all TCGA datasets and achieved remarkable results. On the COADREAD dataset, our model obtained a C-index of 83.2%; on the BRCA dataset, it was 76.5%; on the BLCA dataset, 64.6%; on the HNSC dataset, 66.6%; and on the STAD dataset, 69.8%. Compared with the current state-of-the-art single-modality methods, our method has an improvement of 7.3% on COADREAD, 6.6% on BRCA, 1.8% on BLCA, 3.0% on HNSC, and 4.6% on STAD. This comparison result fully demonstrates the unique advantages of multimodal methods in survival prediction.

**Multimodal baselines.** We conducted a comprehensive comparison of current SOTA multimodal survival prediction methods, including MCAT [9], MOTCAT [40],Porpoise [41], and CMTA [29] with two common late fusion strategies—Cascade (Cat) and Kronecker product (KP), to effectively integrate WSI features with genomic features. We compared the performance of these models with two state-of-the-art multimodal survival prediction methods, PIBD [3] and SurvPath [15].

**Unimodal vs multimodal**. Compared to MLP, SNN, and SNNTrans, which focus on genomic data analysis, the DSCASurv model demonstrated superior performance in all types of benchmark tests, with overall C-index improvements of 11.9, 9.5, and 7.9%, respectively. Additionally, DSCASurv outperformed all pathology-based unimodal methods, with overall C-index performance improvements ranging from 4.7 to 12.7%. This series of comparative results further reveals the immense potential of multimodal data in enhancing survival prediction accuracy.

**Multimodal SOTA vs DSCASurv**. The DSCASurv model not only outperformed a range of leading methods, including PIBD and SurvPath, in terms of multimodal learning and prediction but also achieved a 2.3 to 5.9% improvement in overall performance compared to multimodal methods using fusion mechanisms like Cat and KP. This notable improvement underscores the efficacy of our proposed multimodal integration approach and further consolidates its leading position in survival prediction technology.

### Ablation study

In this section, we conduct some additional experiments to further discuss the impact of different alignment loss, components, and constraints.

**1) Impacts of alignment loss**. We utilized the L1 loss as our default similarity metric. To thoroughly evaluate the effect of different metrics on model performance, we expanded our experiments to include comparisons with mean squared error (MSE) loss and Kullback–Leibler (KL) divergence. The results are visually presented in Table 3. As observed, L1 loss consistently outperforms other metrics in most cases, with the exception of the BRCA dataset.

**Table 3:** Performance under different similarity metrics

**Loss**	**COADREAD**	**BRCA**	**BLCA**	**HNSC**	**STAD**	**Overall**
KL	0.807 $\pm $ 0.093	**0.815 $\pm $ 0.093**	0.642 $\pm $ 0.033	0.640 $\pm $ 0.063	0.695 $\pm $ 0.072	0.720
MSE	0.805 $\pm $ 0.103	0.752 $\pm $ 0.072	0.630 $\pm $ 0.039	0.646 $\pm $ 0.065	0.686 $\pm $ 0.088	0.704
L1	**0.832 $\pm $ 0.095**	0.765 $\pm $ 0.061	**0.646 $\pm $ 0.034**	**0.666 $\pm $ 0.071**	**0.698 $\pm $ 0.093**	**0.721**

**2) Impacts of modules.** We conduct ablation experiments by systematically removing multiple key modules and constraints to evaluate their impact on model performance. The experimental results are summarized in Table 4. Furthermore, a detailed analysis of the effects of missing cross modules, Bi-Mamba modules, IDConv modules, STE modules, PCA modules, and GCA modules can be found in Supplementary Information A.1.

(1) *No alignment module:* When the alignment constraint $ L_{sim} $ was removed, the model’s performance decreased across all datasets. Specifically, the performance dropped by 3.10% on COADREAD, 5.20% on BRCA, 0.50% on both BLCA and STAD, and 4.10% on HNSC, with an overall performance reduction of 2.60%. This indicates that the absence of necessary alignment constraints during the information translation process severely hampers the discriminative ability of intra-modal and cross-modal representations, ultimately weakening the model’s survival prediction performance.
(2) *No CM-Mixer module:* The experimental results indicate that the CM-Mixer module plays a crucial role in the model architecture. Specifically, removing the CM-Mixer module before the cross module led to a decrease in the average C-index to 0.666, while removing the CM-Mixer module after the cross module resulted in a reduction of the average C-index to 0.656. These findings demonstrate that the CM-Mixer module is indispensable for integrating local and global information and enhancing the model’s generalization capability. Its absence severely disrupts the model’s information processing mechanism, significantly impairing the overall performance of the model.

**Table 4:** The experimental results after removing the following key components

**Module**	**COADREAD**	**BRCA**	**BLCA**	**HNSC**	**STAD**	**Overall**
no_Alignment	0.801 $\pm $ 0.095	0.713 $\pm $ 0.069	0.641 $\pm $ 0.036	0.625 $\pm $ 0.067	0.693 $\pm $ 0.068	0.695
no_IDConv	0.779 $\pm $ 0.128	0.714 $\pm $ 0.111	0.629 $\pm $ 0.038	0.624 $\pm $ 0.053	**0.707 $\pm $ 0.062**	0.691
no_Bi-Mamba	0.794 $\pm $ 0.144	0.741 $\pm $ 0.068	0.623 $\pm $ 0.057	0.604 $\pm $ 0.048	0.677 $\pm $ 0.031	0.688
no_Cross	0.773 $\pm $ 0.105	0.723 $\pm $ 0.075	0.658 $\pm $ 0.065	0.648 $\pm $ 0.065	0.682 $\pm $ 0.045	0.697
no_PCA	0.812 $\pm $ 0.117	0.741 $\pm $ 0.046	0.628 $\pm $ 0.053	0.601 $\pm $ 0.059	0.676 $\pm $ 0.046	0.692
no_GCA	0.783 $\pm $ 0.123	0.724 $\pm $ 0.062	0.679 $\pm $ 0.062	0.628 $\pm $ 0.050	0.669 $\pm $ 0.041	0.697
no_STE	0.763 $\pm $ 0.120	0.713 $\pm $ 0.068	0.629 $\pm $ 0.058	0.615 $\pm $ 0.076	0.656 $\pm $ 0.071	0.675
no_CM-Mixer1	0.754 $\pm $ 0.175	0.705 $\pm $ 0.062	0.601 $\pm $ 0.056	0.631 $\pm $ 0.103	0.639 $\pm $ 0.054	0.666
no_CM-Mixer2	0.764 $\pm $ 0.100	0.673 $\pm $ 0.109	0.601 $\pm $ 0.084	0.572 $\pm $ 0.085	0.669 $\pm $ 0.096	0.656
ALL	**0.832 $\pm $ 0.095**	**0.765 $\pm $ 0.061**	**0.646 $\pm $ 0.034**	**0.666 $\pm $ 0.071**	0.698 $\pm $ 0.093	**0.721**

**3) Impacts of modality alignment**. To verify the effectiveness of intra-modal and inter-modal alignment, we examined the impact of different alignment strategies, as shown in Table 5. When the alignment module was completely removed (no_Alignment), the overall C-index dropped by 2.6% compared to the dual-direction alignment (ALL) configuration.With only intra-modal alignment ($g \longleftrightarrow \hat{g}$, $h \longleftrightarrow \hat{h}$), the overall C-index was 1.8% lower than that of dual-direction alignment; using only cross-modal alignment ($g \longleftrightarrow h$, $\hat{g} \longleftrightarrow \hat{h}$), the C-index decreased by 2.2%. These findings indicate that both intra-modal and cross-modal alignment contribute positively to performance, but dual-direction alignment achieves the best predictive accuracy across all datasets. Table 6 summarizes the impact of feature combinations on predictive performance across multiple cancer types. More detailed explanations are provided in Supplementary Information A.2.

**Table 5:** Performance under different modality alignment

**Module**	**COADREAD**	**BRCA**	**BLCA**	**HNSC**	**STAD**	**Overall**
no_Alignment	0.801 $\pm $ 0.095	0.713 $\pm $ 0.069	0.641 $\pm $ 0.036	0.625 $\pm $ 0.067	0.693 $\pm $ 0.068	0.695
$g \longleftrightarrow \hat{g}$ , $h \longleftrightarrow \hat{h}$	0.804 $\pm $ 0.115	0.748 $\pm $ 0.069	0.633 $\pm $ 0.034	0.624 $\pm $ 0.061	**0.705 $\pm $ 0.076**	0.703
$g \longleftrightarrow h$ , $\hat{g} \longleftrightarrow \hat{h}$	0.790 $\pm $ 0.133	0.744 $\pm $ 0.065	**0.660 $\pm $ 0.052**	0.635 $\pm $ 0.052	0.668 $\pm $ 0.049	0.699
ALL	**0.832 $\pm $ 0.095**	**0.765 $\pm $ 0.061**	0.646 $\pm $ 0.034	**0.666 $\pm $ 0.071**	0.698 $\pm $ 0.093	**0.721**

**Table 6:** Performance under different feature combinations

**Feature**	**COADREAD**	**BRCA**	**BLCA**	**HNSC**	**STAD**	**Overall**
$h$	0.710 $\pm $ 0.206	0.709 $\pm $ 0.033	0.625 $\pm $ 0.026	0.611 $\pm $ 0.070	0.681 $\pm $ 0.059	0.667
$\hat{h}$	0.723 $\pm $ 0.158	0.721 $\pm $ 0.055	0.640 $\pm $ 0.061	0.598 $\pm $ 0.049	0.586 $\pm $ 0.082	0.654
$g$	0.663 $\pm $ 0.192	0.622 $\pm $ 0.070	0.583 $\pm $ 0.045	0.544 $\pm $ 0.021	0.596 $\pm $ 0.092	0.602
$\hat{g}$	0.698 $\pm $ 0.165	0.657 $\pm $ 0.076	0.622 $\pm $ 0.043	0.631 $\pm $ 0.060	0.664 $\pm $ 0.080	0.654
$h+g$	0.769 $\pm $ 0.176	0.758 $\pm $ 0.082	0.630 $\pm $ 0.033	0.583 $\pm $ 0.057	0.661 $\pm $ 0.069	0.680
$\hat{h}+\hat{g}$	0.770 $\pm $ 0.143	0.732 $\pm $ 0.067	0.602 $\pm $ 0.038	0.646 $\pm $ 0.045	0.646 $\pm $ 0.079	0.679
$g+\hat{g}$	0.784 $\pm $ 0.126	0.726 $\pm $ 0.092	0.575 $\pm $ 0.058	0.641 $\pm $ 0.033	0.650 $\pm $ 0.079	0.675
$h+\hat{h}$	0.773 $\pm $ 0.159	0.688 $\pm $ 0.092	0.610 $\pm $ 0.056	0.612 $\pm $ 0.054	0.658 $\pm $ 0.053	0.668
ALL	**0.832 $\pm $ 0.095**	**0.765 $\pm $ 0.061**	**0.646 $\pm $ 0.034**	**0.666 $\pm $ 0.071**	**0.698 $\pm $ 0.093**	**0.721**

**4) Impacts of Alpha constraints**. To assess the impact of the Alpha parameter on the performance of the DSCASurv framework, we conducted an ablation study with different Alpha values (0, 0.01, 0.03, 0.1), as shown in Table 7. As Alpha increased, model performance peaked at Alpha = 0.03, with a C-index of 0.721. Therefore, Alpha = 0.03 was chosen as the default value in this study to optimize the accuracy of survival prediction. $\alpha $ is the weighting coefficient for modal alignment, determining the model’s emphasis on different modal information. When $\alpha $ is large, the model focuses more on modal alignment constraints, helping capture potential correlations and complementary information between modalities; when $\alpha $ is small, the model places more focus on the survival prediction task. By adjusting $\alpha $, the model can balance multiple factors, optimizing both survival prediction and modal alignment accuracy. Additionally, an appropriate $\alpha $ value prevents the model from over-relying on specific features or data patterns, improving generalization and better adapting to new data and tasks.

**Table 7:** Performance under different Alpha constraints

**Alpha**	**COADREAD**	**BRCA**	**BLCA**	**HNSC**	**STAD**	**Overall**
0	0.801 $\pm $ 0.095	0.713 $\pm $ 0.069	0.641 $\pm $ 0.036	0.625 $\pm $ 0.067	0.693 $\pm $ 0.068	0.695
0.01	0.794 $\pm $ 0.142	**0.773 $\pm $ 0.072**	0.640 $\pm $ 0.030	0.632 $\pm $ 0.051	0.662 $\pm $ 0.077	0.700
0.03	**0.832 $\pm $ 0.095**	0.765 $\pm $ 0.061	0.646 $\pm $ 0.034	**0.666 $\pm $ 0.071**	**0.698 $\pm $ 0.093**	**0.721**
0.1	0.787 $\pm $ 0.113	0.757 $\pm $ 0.056	**0.658 $\pm $ 0.052**	0.630 $\pm $ 0.087	0.719 $\pm $ 0.036	0.710

### Survival analysis

**Figure 2:**
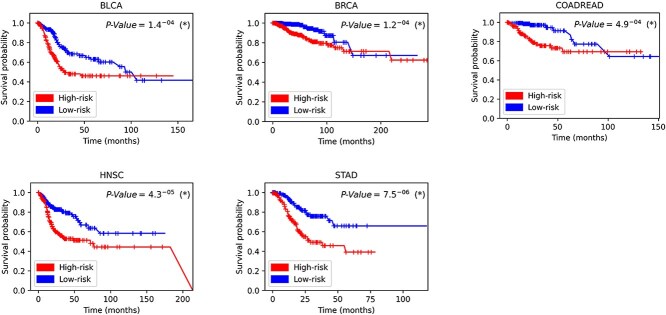
Kaplan–Meier survival curves and log-rank test results.

For further validation to deeply validate the effectiveness of DSCASurv model in survival analysis, we first accurately categorized the patients into two subsets, low-risk and high-risk, based on the median risk score predicted by the model. Subsequently, we employed Kaplan–Meier survival analysis, a classic and intuitive tool that helped us visualize the distribution of the two groups of patients in terms of survival time, and the results of the analysis are detailed in Fig. 2. In addition, we applied the log-rank test, which is a well-recognized statistic for assessing the difference in survival probability between high-risk groups (denoted in red) and low-risk groups (denoted in blue). indicated) whether the difference in survival probability was statistically significant. Typically, a *P*-value of less than 0.05 is considered significant. It is clear from the graph that the *P*-value between the two groups is significantly lower than 0.05 in all datasets, indicating that the risk scores are able to differentiate significantly between the prognosis of the patients.

### Interpretability and attention visualization

**Figure 3:**
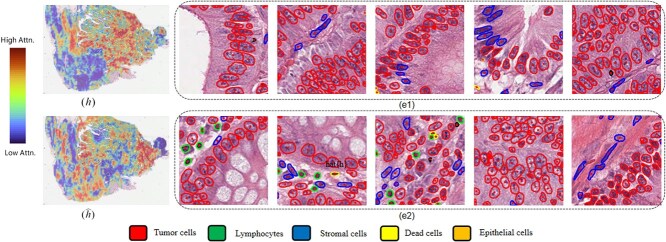
Interpretability and attention visualization.

The DSCASurv model effectively integrates genomic data and pathological image features through a cross-modal attention mechanism, significantly enhancing the model’s interpretability and predictive accuracy. The attention map based on single-modality pathological features (Fig. 3(h)) focuses on cell morphology and tissue structure, while the cross-modal attention map guided by genomic data (Fig. 3($\hat{h}$)) highlights regions closely related to gene expression. This comparison demonstrates that DSCASurv not only captures gene expression-related features but also identifies other diagnostically valuable pathological information, achieving synchronized focus on both pathological and genomic features.Figure 3(e1) and (e2) further showcases the tissue classification results within high-attention regions in the corresponding pathological images. Using HoverNet [42], the model classifies different cell types, such as tumor cells, lymphocytes, and stromal cells. This visualization analysis demonstrates that DSCASurv achieves significant performance improvements in multimodal survival analysis and shows strong potential for clinical applications.

## Conclusion

In an in-depth exploration of the fusion potential between pathology images and genomic data, we propose a novel survival analysis approach through the development of an advanced cross-modal fusion and alignment framework, DSCASurv. This framework employs two parallel and efficient mixer modules to comprehensively mine the rich features within pathology and genomic data, integrating these into highly representative cross-modal feature vectors. On five cancer datasets from TCGA, DSCASurv demonstrates significant superiority over existing survival prediction methods, achieving an average C-Index improvement of 2.3 to 12.7%.Unlike conventional approaches, DSCASurv combines both global and local features, effectively addressing the limitations of methods that focus solely on global information while overlooking local detail, thus better accommodating fine-grained classification tasks. Additionally, DSCASurv leverages a cross-modal attention mechanism to facilitate intra- and inter-modal information exchange and deep integration, substantially enhancing the accuracy and generalization capabilities of survival predictions.

**Key Points**

- This work represents the first successful combination of the Mamba model, convolution, and attention mechanisms for multimodal survival prediction, effectively addressing the challenges of high-dimensional WSIs and transcriptomic data with impressive performance.
- A novel token mixer, named CM-Mixer, is introduced to aggregate sparse global information and local details in a contextsensitive manner, generating strong inductive biases that enhance the model’s generalization capability.
- An innovative cross-modal fusion alignment framework is presented, significantly improving model performance by aligning and harmonizing features across different modalities.
- Extensive experiments conducted on five public TCGA datasets validate the effectiveness of the proposed model. Results demonstrate superior performance compared to state-of-the-art methods.

## Supplementary Material

Supplementary_Information_bbaf103

## Data Availability

TCGA datasets can be accessed through the NIH Genomic Data Commons Data Portal (https://portal.gdc.cancer.gov/). Hallmark pathway analysis resources are available at MSigDB (https://www.gsea-msigdb.org/). Reactome pathway datasets are obtainable through the Reactome platform (https://reactome.org/).
