# Supplementary material for: Dual-stream cross-modal fusion alignment network for survival analysis
Source: Brief Bioinform. 2025 Mar 21;26(2):bbaf103. doi: 10.1093/bib/bbaf103 (PMC11926988; doi:10.1093/bib/bbaf103)
Supplement: Supplementary_Information_bbaf103 [file supplementary_information_bbaf103.docx]

# Supplementary Information

**A Ablation Study**

**A.1 Impacts of Modules.**

**(1)** **No Bi-Mamba Module:** Through the ablation experiment of the Bi-Mamba module, we found that after removing this module, the performance of the model significantly declined on all datasets. On the COADREAD dataset, the C-index dropped from 0.832 of the complete model to 0.794; on the BRCA dataset, it decreased from 0.765 to 0.741. The overall average C-index dropped to 0.688, a 4.6% decrease compared to the original model. This result fully demonstrates that the Bi-Mamba module plays a crucial role in capturing long-range dependencies and integrating global information. Its absence makes it difficult for the model to effectively extract key features from the data, thus affecting the accuracy of survival prediction.

**(2) No IDConv Module:** The IDConv module plays a crucial role in enhancing the model's representation by dynamically aggregating local features and injecting inductive bias. After removing this module, the performance dropped by 5.30% on COADREAD, 5.10% on BRCA, 1.70% on BLCA, and 4.20% on HNSC. Interestingly, a slight performance increase of 0.90% was observed on STAD. Overall, the performance decreased by 3.00%. This suggests that the IDConv module is essential for handling local features effectively, some datasets like STAD may not rely heavily on this mechanism.

**(3) No STE Module:** The STE module plays an important role in enabling effective communication between channels. After its removal, the model’s performance dropped by 4.60% in overall. This demonstrates the STE module’s vital role in ensuring effective channel communication and improving model performance

**(4)No PCA Module:** When the PCA module was removed, the performance of the model changed significantly on all datasets. From the experimental results, on the COADREAD dataset, the C-index dropped from 0.832 of the complete model to 0.812; on the BRCA dataset, it decreased from 0.765 to 0.741; on the BLCA dataset, it dropped from 0.646 to 0.628; on the HNSC dataset, it decreased from 0.666 to 0.601; on the STAD dataset, it dropped from 0.698 to 0.676. The overall performancedropped to 0.692, a 2.9% decrease compared to the complete model. This shows that the PCA module plays an important role in guiding the aggregation of genomic features using pathological information and thus mining the potential relationships between multimodal data. The absence of the PCA module will affect the effective fusion of pathological and genetic information by the model, thereby reducing the ability to predict the survival of patients.

**(5) No GCA Module:** After removing the GCA module, the model performance was also affected. On the COADREAD dataset, the C-index was 0.783; on the BRCA dataset, it was 0.724; on the BLCA dataset, it was 0.679; on the HNSC dataset, it was 0.628; on the STAD dataset, it was 0.669. The overall performance dropped to 0.697, a 2.4% decrease compared to the complete model. This indicates that the GCA module is crucial in guiding the aggregation of pathological features using genomic information and promoting cross-modal information transfer and fusion. After removing the GCA module, the model cannot fully utilize genomic data to optimize pathological features, thus affecting the accuracy of the final survival prediction.

**(6) No Cross Module:** Removing the cross-modal attention module also resulted in a significant performance drop. The overall performance of the model decreased by 2.40%. These results highlight the importance of cross-modal attention in cross-modal interaction and complementary information transfer, thereby enhancing the model's ability to make accurate predictions.

**A.2 Impacts of modality alignment**

Table 6 summarizes the impact of feature combinations on predictive performance across multiple cancer types. Single features showed limited generalizability, with h (0.667) performing best, while g had the lowest score (0.602). Pairwise combinations improved performance but remained inferior to full integration, with h+g achieving a C-index of 0.680 but still falling short overall. The ALL configuration consistently outperformed others across cancer types, particularly in COADREAD (0.832) and BRCA (0.765), demonstrating the benefits of multimodal fusion in enhancing robustness and reducing variance. These findings highlight the necessity of holistic feature integration, with ALL achieving an average improvement of 3.8–11.9% over single features, effectively addressing the challenges of heterogeneous cancer data.
